# Abandoning Presumptive Antimalarial Treatment for Febrile Children Aged Less Than Five Years—A Case of Running Before We Can Walk?

**DOI:** 10.1371/journal.pmed.1000015

**Published:** 2009-01-06

**Authors:** Mike English, Hugh Reyburn, Catherine Goodman, Robert W Snow

## Abstract

Background to the debate: Current guidelines recommend that all fever episodes in African children be treated presumptively with antimalarial drugs. But declining malarial transmission in parts of sub-Saharan Africa, declining proportions of fevers due to malaria, and the availability of rapid diagnostic tests mean it may be time for this policy to change. This debate examines whether enough evidence exists to support abandoning presumptive treatment and whether African health systems have the capacity to support a shift toward laboratory-confirmed rather than presumptive diagnosis and treatment of malaria in children under five.

## Abstract 2

Mike English and colleagues argue that we do not have sufficient evidence and health system capacity to abandon presumptive antimalarial treatment in children with fever in sub-Saharan Africa, countering the argument of Blaise Genton and colleagues in a related paper.

*In this Viewpoint, Mike English and colleagues argue against abandoning presumptive treatment for under-fives. Blaise Genton and colleagues present the opposing Viewpoint in a related article: D'Acremont V, Lengeler C, Mshinda H, Mtasiwa D, Tanner M, et al. (2009) Time to move from presumptive malaria treatment to laboratory-confirmed diagnosis and treatment in African children with fever. PLoS Med 6(1): e252. doi: 10.1371/journal.pmed.0050252*

No one would argue against the tenet that children in low-income settings should receive the highest quality of clinical care. System-wide provision of accurate and reliable treatment to those with true malaria is a major goal. However, we caution against rapid universal policy change that abandons presumptive antimalarial treatment for African children under five with fever for two reasons. Firstly, important evidence gaps remain. Secondly, the health system capacity to implement such a policy shift has not been demonstrated. If anxiety about drug costs (which are falling) and optimism that malaria is being defeated drive rapid policy change, this may result in hurried policy doing more harm than good.

Minimising the risk of death or severe disease is at the heart of the presumptive treatment strategy. Diagnostics potentially allow treatment to be restricted to those truly with disease. However, where diagnostics, or their use, are imperfect, we remain with a (now untreated) population at risk. While rapid diagnostic tests (RDTs) perform relatively well in research studies, limited data on performance in routine settings suggest relatively poor sensitivity overall (65%) and worrying variability between sites (19%–86% sensitivity) [1]. How great a risk are we prepared to take in terms of mortality and morbidity from missed malaria cases? Recent economic evaluations support treatment contingent on RDT diagnosis, particularly where malaria prevalence is low [2,3]. However, such models still rely on a “best guess” of the risk of no treatment in a truly infected child. Furthermore, this best guess often assumes infection in a semi-immune child. This scenario will no longer be relevant as infection rates decline because even young children will be “non-immune” and potentially at much higher risk. Limited data suggest that the risk of failure to detect true malaria in febrile children is very low [4]. However, these studies are based on active follow-up, and do not measure the risks of serious morbidity and mortality from a failed diagnostic process in real-life settings where there are considerable barriers to accessing (re-)treatment.

Malaria prevalence has declined in some countries. However, malaria prevention efforts across the continent remain inadequate [5], and considerable heterogeneity persists within countries, with relatively high prevalence (more than 40%) recently reported in febrile under-fives in Tanzania and Uganda [1,6,7]. Ideally, countries would base their diagnostic strategy on detailed data on local epidemiology and disease risks [8]; however, most countries simply do not have the right information, and may lack the capacity to vary diagnostic strategies by transmission level.

The beliefs and behaviours of health workers and patients are central to improving the rational use of antimalarials. In operational settings health workers frequently ignore diagnostic results and prescribe antimalarials to negative cases [9] for reasons that seem to them entirely justified [10]. This behaviour significantly impairs the cost-effectiveness of introducing confirmed diagnosis [3,11]. Furthermore, presumptive treatment by community health workers and retailers is increasingly being promoted with the justifiable aim of improving child survival by increasing access to care. However, a policy context that restricts antimalarials to confirmed diagnoses in formal care settings but promotes presumptive treatment in the community will send mixed messages to patients and health workers, and reinforce the view that all fever (and almost all non-specific illness) is “malaria”. A negative diagnostic test may do little to change this view [9,10].

Moreover, introducing universal diagnostic testing is not a matter of behaviour change alone. There are also major health system challenges. We have proven ourselves incapable of even purchasing and delivering drugs reliably [12]. Adding a new commodity that requires distribution and quality control [1] will be a considerable challenge, and arguably one that should be taken on only once we have achieved high coverage with effective drugs and prevention.

Improving diagnosis and treatment of febrile illness is a key priority. However, before embarking on a universal policy change we need: (1) improved data on local malaria epidemiology, perhaps obtained initially through specific sentinel sites; (2) improved implementation of confirmed diagnosis and treatment in older children and adults, specifically tackling health worker and patient behaviours and beliefs; (3) information on the safety and acceptability of withholding antimalarials in young febrile children who test negative by RDT for malaria; and (4) development of effective quality control processes to support RDT introduction. Much of this work could be undertaken as part of pragmatic, large-scale effectiveness assessments in areas already making excellent progress in controlling malaria. While such work is undertaken we must not lose sight of the necessity to reduce current, inequitable gaps in coverage with established malaria prevention and treatment strategies and primary health care more broadly.

## English and Colleagues' Response to Genton and Colleagues' Viewpoint:

*In this section, English and colleagues respond to the points raised by Genton and colleagues in their opposing Viewpoint: D'Acremont V, Lengeler C, Mshinda H, Mtasiwa D, Tanner M, et al. (2009) Time to move from presumptive malaria treatment to laboratory-confirmed diagnosis and treatment in African children with fever. PLoS Med 6(1): e252. doi: 10.1371/journal.pmed.0050252*

Genton and colleagues present the case for a shift from presumptive antimalarial treatment for all febrile children in malaria-endemic areas to a policy of treatment based on confirmatory diagnostic testing with an RDT. The logic of this argument is that: (1) we have the diagnostic tools; (2) drugs are costly, and unnecessary treatment exposes children to needless risks; (3) malaria prevalence has declined sufficiently so universal treatment is neither clinically nor economically rational; and (4) over-diagnosis of malaria results in inadequate treatment of other illnesses. Implicit in their suggestion for a comprehensive policy change now is that health systems and health workers are ready for it. As we have already described in our Viewpoint, there is very little published data on RDT use in real-life settings and what there is suggests their performance is currently far from convincing. And while we agree that malaria prevalence is declining in many areas, we maintain that malaria epidemiology varies considerably and individual countries lack adequate information on local epidemiology and disease risk to meaningfully tailor their diagnostic strategies by transmission level.

Perhaps the most important barriers to improving the rational use of antimalarials are the limited capacities of health systems and health workers. Genton and colleagues imply that changing the mindset of health workers and patients will be relatively straightforward. It is more likely that making it acceptable to “deny” a patient (especially a young, febrile child) an antimalarial will be a mammoth task. After all, we have made no progress in reducing the use of antimalarials to date even in adults in low-transmission settings—perhaps a useful place to begin rigorously implementing a policy of treatment based on a positive diagnosis, as this group represents a large fraction of drug costs.

Furthermore, Genton and colleagues argue (but provide no evidence) that identifying cases negative for malaria will improve overall quality of care. There is in fact little reassurance that availability of malaria tests makes health workers better diagnosticians, as they often ignore all guidelines and follow norms that are not obviously rational [10] but may reflect “bedside rationing” in severely resource-constrained environments. Indeed, the current Integrated Management of Childhood Illness approach already encourages assessment and treatment of all likely diagnoses in under-fives irrespective of the presence of malaria. We cannot blame the provision of poor quality primary care on the lack of malaria diagnostics, and it seems unlikely that their provision will automatically improve availability of appropriate resources for treating other conditions and improving care more generally. Such general improvement needs to be addressed as a priority in its own right.

## Abbreviations

RDT: rapid diagnostic test
